# Safety, recommended dose, efficacy and immune correlates for nintedanib in combination with pembrolizumab in patients with advanced cancers

**DOI:** 10.1186/s13046-022-02423-0

**Published:** 2022-07-07

**Authors:** Capucine Baldini, Francois-Xavier Danlos, Andreea Varga, Matthieu Texier, Heloise Halse, Severine Mouraud, Lydie Cassard, Stéphane Champiat, Nicolas Signolle, Perrine Vuagnat, Patricia Martin-Romano, Jean-Marie Michot, Rastislav Bahleda, Anas Gazzah, Lisa Boselli, Delphine Bredel, Jonathan Grivel, Chifaou Mohamed-Djalim, Guillaume Escriou, Laetitia Grynszpan, Amelie Bigorgne, Saloomeh Rafie, Alae Abbassi, Vincent Ribrag, Sophie Postel-Vinay, Antoine Hollebecque, Sandrine Susini, Siham Farhane, Ludovic Lacroix, Aurelien Parpaleix, Salim Laghouati, Laurence Zitvogel, Julien Adam, Nathalie Chaput, Jean-Charles Soria, Christophe Massard, Aurelien Marabelle

**Affiliations:** 1 Département d’Innovation Thérapeutique et d’Essais Précoces (DITEP), Gustave Roussy, Villejuif, France; 2 INSERM U1015 & CIC1428, Gustave Roussy, Villejuif, France; 3 Faculté de Médecine, Université Paris Saclay, Le Kremlin-Bicetre, France; 4 Département de Biostatistiques, Gustave Roussy, Villejuif, France; 5 INSERM U1163, Institut Imagine, Paris, France; 6 Laboratoire d’Immuno-Oncologie (LIO), CNRS-UMS 3655 and INSERM-US23, Gustave Roussy, Villejuif, France; 7 INSERM U981, Department of Experimental Pathology, Gustave Roussy, Université Paris-Sud, Université Paris-Saclay, 94805 Villejuif, France; 8 Département d’Oncologie Médicale, Institut Curie, Paris, France; 9 INSERM UMR 1186, Integrative Tumor Immunology and Immunotherapy, Gustave Roussy, Faculté de Médecine, Université Paris-Sud, Université Paris-Saclay, 94805 Villejuif, France; 10 INSERM U981, Gustave Roussy, Villejuif, France; 11 Département de Biopathologie, AMMICA, INSERM US23/CNRS UMS3655, Gustave Roussy, Université Paris-Saclay, Villejuif, France; 12 Service de Promotion des Etudes Cliniques, Gustave Roussy, Villejuif, France; 13 Unité Fonctionnelle de Pharmacovigilance, Villejuif, France; 14 Service d’Anatomo-Pathologie, Hôpital Paris Saint-Joseph, Paris, France; 15 Amgen, Thousand Oaks, CA USA

**Keywords:** Phase I, Dose escalation, Immunotherapy, Anti PD-1, Anti-angiogenic

## Abstract

**Background:**

We aimed to determine the safety and efficacy of nintedanib, an oral anti-angiogenic tyrosine kinase inhibitor, in combination with pembrolizumab, an anti-PD1 immunotherapy, in patients with advanced solid tumors (PEMBIB trial; NCT02856425).

**Methods:**

In this monocentric phase Ib dose escalation cohort, we evaluated escalating doses of nintedanib (Dose level 1 (DL1) = 150 mg bid [*bis in die*, as twice a day]; DL2 = 200 mg bid, oral delivery) in combination with pembrolizumab (200 mg Q3W, IV). Patients received a 1-week lead-in dose of nintedanib monotherapy prior starting pembrolizumab. The primary objective was to establish the maximum tolerated dose (MTD) of the combination based on dose limiting toxicity (DLT) occurrence during the first 4 weeks. Secondary objectives were to assess the anti-tumor efficacy and to identify the associated immune and angiogenic parameters in order to establish the recommended nintedanib dose for expansion cohorts. Flow cytometry (FC), Immuno-Histo-Chemistry (IHC) and electrochemiluminescence multi-arrays were prospectively performed on baseline & on-treatment tumor and blood samples to identify immune correlates of efficacy.

**Results:**

A total of 12/13 patients enrolled were evaluable for DLT (1 patient withdrew consent prior receiving pembrolizumab). Three patients at 200 mg bid experienced a DLT (grade 3 liver enzymes increase). Four patients developed grade 1–2 immune related adverse events (irAE). Eight patients died because of cancer progression. Median follow-up was 23.7 months (95%CI: 5.55–40.5). Three patients developed a partial response (PR) (ORR = 25%) and five patients (42%) had durable clinical benefit (DCB), defined as PR or stable disease (SD) ≥ 6 months. At baseline, patients with DCB had higher plasma levels of Tie2, CXCL10, CCL22 and circulating CD4^+^ PD1^+^ OX40^+^ T cells than patients without DCB. Patients with DCB presented also with more DC-LAMP^+^ dendritic cells, CD3^+^ T cells and FOXP3^+^ Tregs in baseline tumor biopsies. For DCB patients, the nintedanib lead-in monotherapy resulted in higher blood CCL3, Tregs and CCR4^+^ CXCR3^+^ CXCR5^−^ memory CD4 T cells. After the first pembrolizumab infusion, patients with DCB showed lower IL-6, IL-8, IL-27 plasma levels.

**Conclusion:**

Nintedanib 150 mg bid is the recommended dose for combination with pembrolizumab and is currently investigated in multiple expansion cohorts. Early tumoral and circulating immune factors were associated with cancer outcome under nintedanib & pembrolizumab therapy.

**Trial registration:**

ClinicalTrials.gov, NCT02856425. Registered August 4, 2016 — Prospectively registered.

**Supplementary Information:**

The online version contains supplementary material available at 10.1186/s13046-022-02423-0.

## Background

Angiogenesis is a key mechanism in tumour growth and development of metastases [1]. Tumours induce blood vessel growth (angiogenesis) by secreting growth factors such as the Vascular Endothelial Growth Factor (VEGF), fibroblast growth factor (FGF) and platelet-derived growth factor (PDGF). VEGF and its high affinity receptor VEGFR-2 are crucial for the formation of new tumour vessels [2]. In addition, there is preclinical evidence that FGF, PDGF and their associated receptor tyrosine kinases substantially contribute to tumour angiogenesis. The VEGF/VEGFR-2 axis may also generate an autocrine loop which stimulates growth of tumour cells [3]. Therefore, suppression of neo-angiogenesis via inhibition of VEGFR-2 is a promising and efficient strategy for the treatment of solid tumors [4].

Immune checkpoint blockade is a new treatment strategy undergoing extensive investigation in multiple malignancies. Unlike standard chemotherapy or targeted therapy, the immune checkpoint blockade restores the immune system’s capacity to eradicate tumours [5]. Antibodies targeting the Programmed Death-1 (PD-1) receptor and its ligand (PD-L1) have been extensively investigated and are still in active development across malignancies and their different lines of treatment [6]. Combining anti-angiogenic drugs and anti-PD-(L)1 therapy has recently shown important synergistic results in renal cell carcinoma (RCC) and hepatocellular carcinoma (HCC) [7–9]. Indeed, anti-VEGF therapies may enhance anti–PD-(L)1 efficacy by reversing VEGF-mediated immunosuppression and promoting T-cell infiltration in tumours [10–15].

Although the combination of anti-angiogenics together with immune checkpoint blockade is becoming an attractive combination for the treatment of many cancers [16], the safety and activity of such combination is depending on the anti-angiogenic molecule tested. For instance, combinations of sunitinib and pazopanib with the anti-PD1 nivolumab were deemed too toxic with more than 70% grade 3/4 treatment related adverse events [17]. Therefore, novel anti-angiogenics should be tested in dedicated clinical trials to establish the dose, regimen, safety and activity when used in combination with anti-PD1 immunotherapies [18]. Nintedanib is an oral small-molecule tyrosine kinase inhibitor (TKI) that has been approved in 2014 by the EMA in combination with docetaxel for the treatment of adult patients with locally advanced, metastatic or locally recurrent non-small cell lung cancer (NSCLC) of adenocarcinoma histology after first-line chemotherapy. It acts as a triple angiokinase inhibitor blocking VEGF receptors (VEGFR 1–3), PDGF receptors (PDGFR α and β) and FGF receptors (FGFR 1–3) kinase activity.

We aimed to determine for the first time in humans the safety and efficacy of nintedanib, in combination with pembrolizumab, a humanized IgG4 anti-PD1 monoclonal antibody immunotherapy in patients with advanced solid tumors (NCT02856425).

## Patients and methods

### Study design

In this monocentric phase Ib dose escalation cohort (NCT02856425), we evaluated escalating doses of nintedanib (Dose level 1 (DL1) = 150 mg bid, DL2 = 200 mg bid) in combination with IV flat dose of pembrolizumab over 30 minutes (200 mg Q3W). Two dose-levels below the approved regimen of nintedanib (DL-1 & DL-2) could also be tested in case of unacceptable toxicities upon combination with pembrolizumab. Patients received a one-week lead-in course of nintedanib monotherapy prior starting pembrolizumab. The nintedanib monotherapy lead-in had two main objectives. First, based on the abundant literature on the positive immune modulatory effects of anti-angiogenic TKIs, we aimed at modifying the tumor microenvironment to maximize the potential effects of anti-PD1 immunnotherapy. Second, we wanted to be in a situation where we could assess separately the respective effects of nintedanib and pembrolizumab by comparing blood samples prior treatment (at D-7), after one week of nintedanib (D1) and upon addition of pembrolizumab (C2D1). The sample size of the dose escalation cohort was conducted according to the rolling 6 design [19]. Up to 6 patients evaluable for dose-limiting toxicity (DLT) could be exposed to a dose level. As soon as 3 patients were evaluable for DLT at a given dose level, dose escalation or de-escalation was permitted upon review of the safety data. The protocol was first approved by the Agence Nationale de Sécurité du Médicament (ANSM) on June 24th, 2016 (Ref #160371A-12) and by the Ethical Committee (Comité de Protection des Personnes Ile-de-France 1) on Jul 12th, 2016 (Ref #2016-mai-14236ND). The trial was first posted on clinicaltrials.gov on Aug 4th 2016 (NCT02856425).

### Patients

Eligible patients had advanced, metastatic cancer which progressed after at least one line of standard therapy or were intolerant to standard therapy, naive to immune checkpoint blockade and nintedanib. Additional inclusion criteria included age ≥ 18 years, Eastern Cooperative Oncology Group (ECOG) performance status of 0–1, adequate organ function, measurable disease according to RECIST v1.1, and written informed consent. Key exclusion criteria were radiographic evidence of cavitary tumors, local invasion of major blood vessels and/or at risk for perforation, history of clinically significant hemoptysis within the past 3 months, history of clinically significant hemorrhagic or thromboembolic event in the past 6 months, history of significant cardiovascular diseases, prior treatment with anti PD-(L)1 agents, concurrent steroid medication, history of autoimmune and inflammatory disease. This study was conducted in compliance with the Declaration of Helsinki and the International Ethical Guidelines for Biomedical Research Involving Human Subjects.

### Procedures

Screening procedures were performed up to 21 days (D-28) before Day − 7 (start of nintedanib). Patients continued treatment until disease progression, undue toxicity, or withdrawal of consent for a maximum duration of 24 months.

Adverse events were graded using National Cancer Institute (NCI) CTCAE Version 4.03. The occurrence of a non-hematological toxicity ≥ CTCAE Grade 3, a hematological toxicity ≥ CTCAE Grade 4, or an inability to resume nintedanib dosing within 7 days of stopping due to treatment related toxicity during the first 4 weeks were considered as a DLT, if judged by the Investigator to be possibly, probably, or related to study drug administration. Tumor evaluation was performed every 6 weeks based on Response Evaluation Criteria in Solid Tumors (RECIST) 1.1 and immune-related RECIST (irRECIST) [20, 21]. Details about screening exams, Nintedanib dose modification criteria, and other patient management rules are detailed in the protocol of the trial provided in the Supplementary information files.

Patients underwent tumor biopsies prior to the beginning of nintedanib and prior to the second injection of pembrolizumab in order to monitor the pharmacodynamic effects of the treatment and to identify factors associated with efficacy.

### Outcomes

The primary objective was to establish the maximal tolerated dose (MTD) of nintedanib in combination with pembrolizumab based on the assessment of DLT occurrence during the first 4 weeks (28 days since C1D1). Secondary objectives were to determine the tolerability and safety of the Recommended Phase 2 Dose (RP2D) of nintedanib combined with pembrolizumab and to evaluate first anti-tumor activity of this combination in expansion cohorts. The aim of the ancillary studies was to identify factors associated with efficacy.

### Tumor mutational burden by circulating tumor DNA sequencing

Plasma Cell-free circulating DNA samples were analysed using Oncomine™ Tumor Mutation Load Assay, according to the manufacturer recommendation, covering 1.65 Mb across 409 genes relevant cancer (Thermo Fisher Scientific, Les Ulis, France). Briefly, circulating the DNA were extracted by Maxwell® RSC ccfDNA Plasma Kit (Promega, Charbonnières-les-Bains, France). Libraries were performed using Ion Chef™ Instrument and Ion 550 Chip, then sequenced with Ion S5™ System (Thermo Fisher Scientific). The median coverage depth 751 X (from 636X to 817X) was obtained. Variant callings and Tumor mutational burden (TMB; count of mutations / megabase) were calculated using Ion reporter software package (version 5.14.1.0) with the pipeline: Oncomine Tumor Mutation Load - w3.1 - DNA - Single Sample (Thermo Fisher Scientific).

### Immuno-Histo-chemistry staining

Multiple chromogenic and one fluorescent multiplex Immuno-Histo-Chemistry (IHC) stainings were performed on each biopsy. The Ventana Discovery Ultra platform was used for both PD-L1 and ICAM1 (DAB) single chromogenic staining, for FOXP3 (Purple)/CD31 (DAB) multiplex and CD3 (Purple)/CD8 (DAB) multiplex chromogenic staining and for a panel combining DC-LAMP (OPAL 620), CD68 (OPAL 570), IDO1 (OPAL 540), CD163 (OPAL 520) and DAPI nuclear staining for the identification of myeloid cells. The multispectral images were captured using the Vectra® microscope tunable filter that shines 35 incremental wave lengths of light from 300 nm to 750 nm. High-powered images were selected by a pathologist, matched to a Hematoxylin and Eosin Staining (HES) to validate tumor-associated tissue. The spectral library was synthesized using the Inform® v2.2 software.

### Immune monitoring – fresh blood immune phenotype

For each patient, heparinized blood samples (30–40 mL) at day − 7 (baseline), C1D1 and C5D1 were collected whenever possible for monitoring circulating immune populations by flow cytometry. Fresh whole blood phenotyping of T-cell migration, T-cell polarization, T-cell activation, Treg function and myeloid cells was performed using 5 specific panels, as previously described [22]. Stained cells were acquired using a Gallios Cytometer (Beckman Coulter) and analyzed using Kaluza software (Beckman Coulter).

### Cytokine, chemokine and soluble angiogenic factors measurements

Plasma samples were centrifuged for 15 min at 1000 g, diluted 1:4, then monitored using the Angiogenesis Panel 1 (human) (Meso Scale Discovery, ref.: K151P3S-1), Chemokines Panel 1, Proinflammatory Panel 1, Cytokine Panel 1 (Meso Scale Discovery, ref.: K151A9H-1), the ultra-sensitive assay S-plex Human IFNα2a kit (Meso Scale Discovery, ref.: K151P3S-1), Human PD-1 and PD-L1 antibody sets (Meso Scale Discovery, ref.: F214A-3 & F214C-3, respectively) following manufacturer’s instructions. Acquisitions and analyses were performed on a MESO™ QuickPlex SQ120 reader and the MSD’s Discovery Workbench 4.0. Each plasma sample was assayed twice with the average value taken as the result.

### Statistical analysis and illustrations

**Fig. 1:**
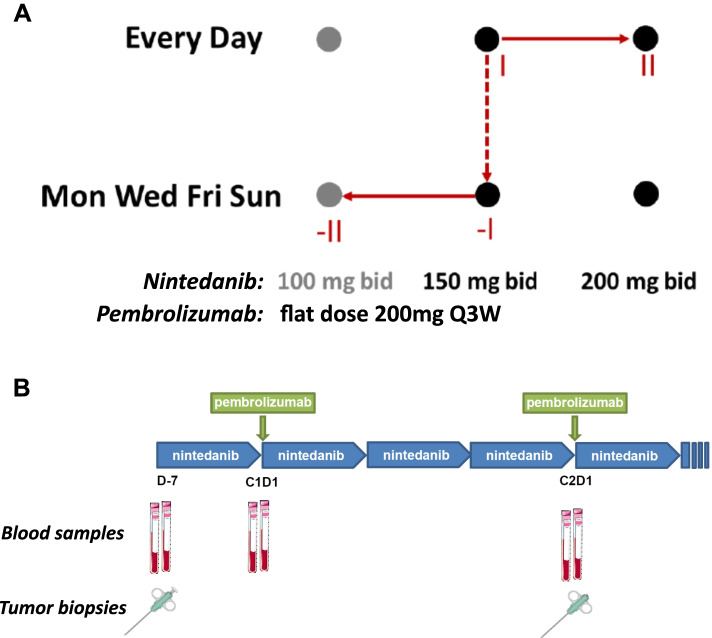
Decision tree for the nintedanib dose escalation (**A**) and the collection of biological samples for ancillary analysis (**B**)

Clinical statistical analysis had been done using the SAS® statistical software version 9.4 (Cary, North Carolina, USA). Calculations and statistical tests for ancillary analyses were performed using R v3.4. Wilcoxon-Mann-Whitney test was used to assess differences between two patient’s groups. Data representation was performed with software R v3.3.3 using tidyverse, dplyr, ggplot2 and ggpubr packages. Figure’s aesthetics were worked with Affinity Designer® (v1.9.2.1035). Figure 1

## Results

### Safety of nintedanib dose escalation in combination with pembrolizumab

Thirteen patients were enrolled between Nov 24th, 2016, and Jul 24th, 2017, in the dose escalation cohort. Among them, one patient withdrew consent before C1D1. Consequently, 12 patients were evaluable for DLT. Six patients received nintedanib 150 mg bid per os (po) and 6 others consecutive patients received nintedanib 200 mg bid po. Their clinical characteristics are described in Table 1. Patients were treated for different tumor types: cervical cancer (*n* = 2), thymic carcinoma (*n* = 2), mesothelioma (*n* = 2), breast cancer (*n* = 1), colorectal adenocarcinoma (*n* = 1), gastric adenocarcinoma (*n* = 1), RCC (*n* = 1), neuroendocrine tumor of the caecum (*n* = 1) and undifferentiated carcinoma of nasopharyngeal type (UCNT) (*n* = 1). All patients received at least one previous line of treatment: chemotherapy (*n* = 12, 100%), immunotherapy (*n* = 1, 8%; intra-tumoral TLR7/8 agonist) and tyrosine kinase inhibitor (*n* = 1, 8%; pazopanib).

**Table 1:** Clinical characteristics of patients enrolled in the dose escalation cohort of the PEMBIB phase 1b trial

	DL1 ***Nintedanib*** ***150 mg bid*** ***N = 6***	DL2 ***Nintedanib*** ***200 mg bid*** ***N = 6***	***Total*** ***N = 12***
Sex			
Male	3 (50%)	3 (50%)	6 (50%)
Female	3 (50%)	3 (50%)	6 (50%)
Age, Median (range)	62 (43–74)	49 (40–67)	59 (40–74)
ECOG PS			
0	3 (50%)	4 (67%)	7 (58%)
1	3 (50%)	2 (33%)	5 (42%)
Breast cancer	0	1 (17%)	1 (8%)
Cervical cancer	2 (33%)	0	2 (17%)
Colorectal carcinoma dMMR	1 (17%)	0	1 (8%)
Gastric carcinoma	1 (17%)	0	1 (8%)
Renal clear cell carcinoma	1 (17%)	0	1 (8%)
Caecal neuroendocrine carcinoma	0	1 (17%)	1 (8%)
Peritoneal mesothelioma	0	1 (17%)	1 (8%)
Pleural mesothelioma	0	1 (17%)	1 (8%)
Thymic carcinoma	0	2 (33%)	2 (17%)
Nasopharyngeal undifferenciated carcinoma	1 (17%)	0	1 (8%)
Median previous lines of treatment	2 (1–5)	1.5 (1–2)	2 (1–5)
*Lung Immune Prognostic Index* (LIPI)			
Good	6 (100%)	2 (33%)	8 (67%)
Intermediate	0	4 (67%)	4 (33%)
Poor	0	0	0
GRIm-score			
0–1	6 (100%)	3 (50%)	9 (75%)
2–3	0	3 (50%)	3 (25%)
Median Neutrophil (.10^3^/mm^3^) (range)	4.45 (3.6–6.1)	3.75 (2–5.4)	4.2 (2.5–6.1)
Median Lymphocytes (.10^3^/mm^3^) (range)	1.4 (1.2–3.2)	1.1 (0.6–1.4)	1.2 (0.6–3.2)
Median Albumin (g/L) (range)	40 (36–44)	42.5 (35–50)	40.5 (35–50)
Median LDH UI/L (range)	186 (147–245)	288 (154–550)	208 (147–550)
Median CRP (mg/L) (range)	11.1 (5.9–68.2)	17.3 (4.9–49.8)	11.1 (4.9–68.2)

Abbreviations: *DL* Dose Level, *ECOG PS* Eastern Cooperative Oncology Group Performance Status, *dMMR* mismatch repair deficient, *LIPI* Lung Immune Prognostic Index [23], *GRIm-score* Gustave Roussy Immune Score [24]

Patients treated with nintedanib 150 mg bid and 200 mg bid received a median of 9 (range: 4;34) and 3 (range: 2;16) complete cycles, respectively. Dose modifications of nintedanib occurred in 2 patients treated with nintedanib 150 mg bid (33%) and 4 patients (67%) and 200 mg bid, respectively. Three patients experienced DLT at 200 mg bid (nintedanib-related liver toxicities). The MTD of nintedanib with pembrolizumab 200 mg Q3W was 150 mg bid, based on DLT occurred in this cohort.

All patients presented at least one adverse event associated with one of both drugs. Four of them developed a defined immune related adverse event, a colitis, a nephritis and two thyroiditis (grade 3, 2 & 1 CTCAE v.4.03, respectively). Superior mesenteric artery occlusion was observed in one patient, concomitantly of a grade 3 colitis, confirmed by biopsies, and a thrombus on catheter in superior vena cava. These events were related to both nintedanib and pembrolizumab. One patient had an acute grade 4 pneumonitis related to *Streptococcus pneumoniae* infection, not related to the experimental combination. Listing of adverse events related to treatments are reported in Table 2.

**Table 2:** Summary of all adverse events reported by investigators in patients treated by nintedanib + pembrolizumab

Nintedanib dose	Grade 1–2		Grade 3–4	
	150 mg BID	200 mg BID	150 mg BID	200 mg BID
Alanine aminotransferase increased	1 (16.7%)	1 (16.7%)	0	3 (50%)
Aspartate aminotransferase increased	1 (16.7%)	0	0	2 (33%)
Diarrhea	3 (50%)	1 (16.7%)	0	0
Hypothyroidism	2 (33%)	1 (16.7%)	0	0
Nausea	2 (33%)	1 (16.7%)	0	0
Vomiting	2 (33%)	1 (16.7%)	0	0
GGT increased	0	1 (16.7%)	0	1 (16.7%)
Fatigue	1 (16.7%)	1 (16.7%)	0	0
Abdominal pain	1 (16.7%)	1 (16.7%)	0	0
Decreased appetite	2 (33%)	0	0	0
Cutaneous rash	2 (33%)	0	0	0
Hypertension	1 (16.7%)	0	0	0
Venous thromboembolism	0	0	1 (16.7%)	0
Colitis	1 (16.7%)	0	0	0
Creatinin increased	0	1 (16.7%)	0	0
Dyspnea	0	1 (16.7%)	0	0
Headache	0	1 (16.7%)	0	0
Hearing impairment	0	1 (16.7%)	0	0
Hyperthyroidism	1 (16.7%)	0	0	0
Mucositis	1 (16.7%)	0	0	0
Nervous system disorder	0	1 (16.7%)	0	0
Peripheral motor neuropathy	1 (16.7%)	0	0	0
Platelet count decreased	1 (16.7%)	0	0	0
Renal and urinary disorder	0	1 (16.7%)	0	0
Supraventricular tachycardia	1 (16.7%)	0	0	0
Weight loss	1 (16.7%)	0	0	0

### Antitumoral activity upon nintedanib dose escalation in combination with pembrolizumab

**Fig. 2:**
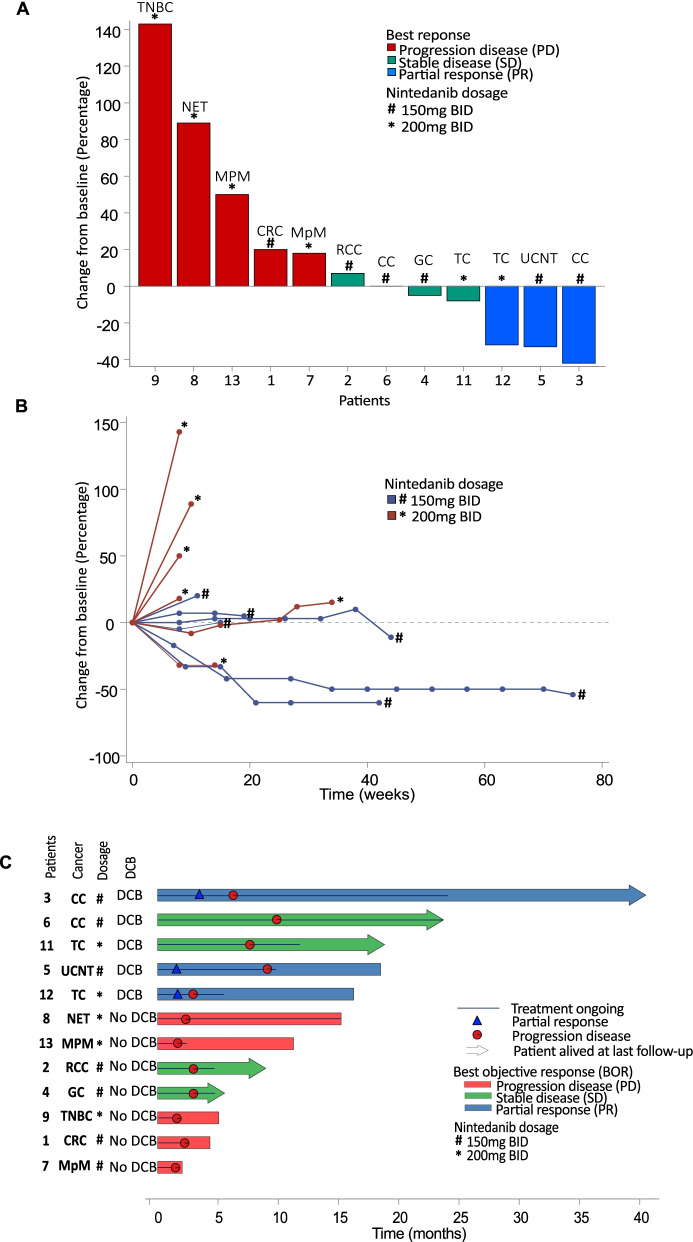
Cancer outcomes in the dose escalation cohort of the PEMBIB trial: waterfall plot (**A**), spider plot (**B**) and swimmer plot (**C**)

Median follow up of the patients was 23.7 months (95% Confidence interval: [5.55; 40.5]). The best objective response was partial response (PR) for 3 patients (25%), stable disease (SD) for 4 patients (33%) and a primary progressive disease (PD) for 5 patients (42%) (Summarized in waterfall plot, Fig. 2A). Two patients with stable disease did not develop a tumor progression during follow up (Fig. 2B). Best responses were achieved at cycle 3 in most cases (58%). Five patients were alive at the end of the follow up period (Figs. 2C). Median Overall Survival was 16.3 months (95% Confidence Interval: [4.34; Not Reached]). Survival rates were 75% (95% CI: [46.8–91]), 64% (95% CI: [35.7–85.4]) and 32% (95% CI: [11.8–62.6]) at 6, 12 and 24 months, respectively. Eight patients died because of cancer progression and no treatment-related deaths were observed. We sequenced the circulating tumor DNA (ctDNA) of our patients in order to better characterize the genomic landscape of our patients’ cancers. No specific relationship could be found between efficacy and the somatic genomic alterations of our patients, including tumor mutational burden (TMB) (Table 3). Of note, TMB from ctDNA were low in this cohort of patients (median 4.19 mutations/Mb [3.35–5.05]) and did not discriminate the patients’ outcomes (Fig. 2C).

**Table 3:** Patient’s genomic landscape by circulating tumor DNA (ctDNA) sequencing

Pt ID	Tumor Histology	DCB	TMB (mut/Mb)	NbVarCalls	Variant (gene; point mutation; variant allelic frequency; coverage)
01	Colorectal carcinoma	No DCB	10,9	13	PIK3CA; p.Glu545Lys; 4%; 900X AKT1; p.Glu17Lys; 4%; 992X GNAS; p.Arg201His; 4%; 299X
02	Renal cell carcinoma	No DCB	2,52	3	NA
03	Cervical cancer	DCB	2,52	3	DNMT3A; p.Phe521SerfsTer24; 25%; 1023X
04	Gastric cancer	No DCB	0,84	1	NA
05	Undifferentiated carcinoma of nasopharyngeal type	DCB	2,51	3	NA
06	Cervical cancer	DCB	0,84	1	NA
07	Mesothelioma	No DCB	5,05	6	PTEN; p.Tyr65Ter; 7%; 408X NOTCH1; p.[Gln2393His;Gln2394Ter]; 16%; 309X
08	Neuroendocrine tumor	No DCB	3,35	4	KRAS; p.Gly12Asp; 25%; 449X TP53; p.Glu343Ter; 53%; 1533X
09	Triple Negative Breast Cancer	No DCB	3,35	4	TP53; p.Trp91Ter; 23%; 714X
11	Thymic carcinoma	DCB	4,19	5	NA
12	Thymic carcinoma	DCB	5,03	6	CDKN2A; p.Val59AlafsTer79; 88%; 1212X TP53; p.Arg282Pro;66%; 1895X
13	Mesothelioma	No DCB	3,32	4	NA

### Preexisting immune & angiogenic characteristics were associated with antitumoral response upon immune checkpoint blockade in combination with antiangiogenic therapy

**Fig. 3:**
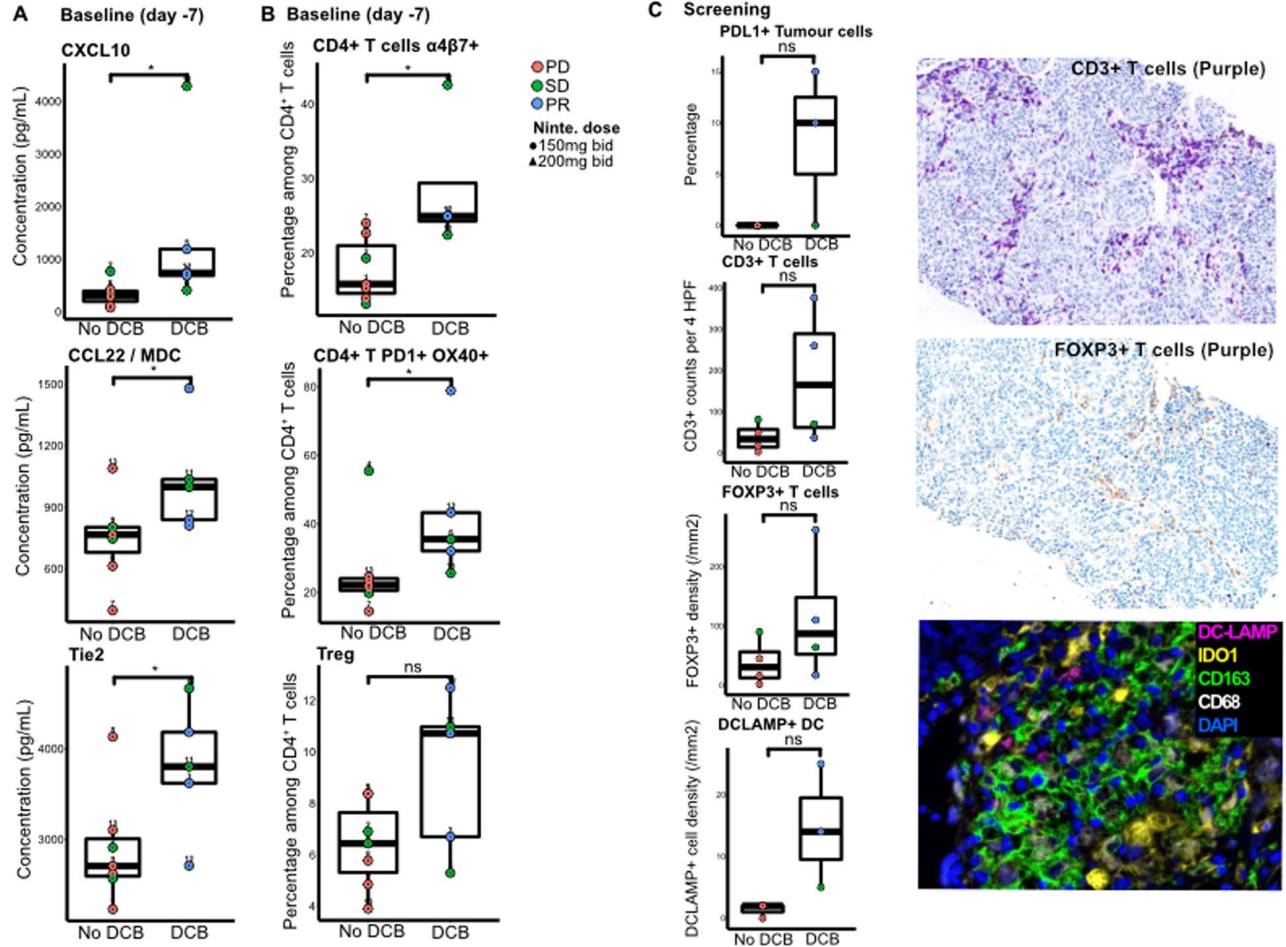
Increased tumor immune cell infiltrations in patients with durable clinical benefit (DCB) before initiation of the nintedanib and pembrolizumab association. **A** CXCL10, CCL22 and soluble Tie2 plasma levels were significantly higher in patients with DCB (1464 pg/mL [range: 409.8–4290.5], 1032.8 pg/mL [range: 810.5–1479.9] and 3796.6 pg/mL [range: 2710.9–4665.6], respectively) than patients without DCB (334.7 pg/mL [range: 81.1–767.6], 745.2 pg/mL [range: 396.8–1088.5] and 2896.5 pg/mL [range: 2230.4–4132.7]) (Wilcoxon rank sum test; *p* = 0.03, *p* = 0.03 and *p* = 0.04, respectively). **B** The percentage of α4β7+ CD4+ and PD1+ OX40+ CD4+ cells among total circulating CD4+ T cells was significantly higher in patients with DCB (28.7% [range: 22.4–42.6] and 43.1% [range: 25.6–78.9], respectively) than without DCB (17.7% [range: 13.1–23.9] and 25.9% [range: 14.5–55.4]) (Wilcoxon rank sum test; *p* = 0.024 and *p* = 0.03, respectively). **C** Patients with PR had PD-L1 expression on tumor cells and immune infiltration in tumor stroma, characterized by IHC (Wilcoxon rank sum test; non-significant). *Abbreviations: PD = Progressive disease; SD = Stable disease; PR = Partial response; Ninte. = nintedanib; DCB = Durable clinical benefit; MDC = Macrophage Derived Chemokine; DC = dendritic cells*

Patients with durable clinical benefit (DCB) defined as objective partial response or stable disease for at least 6 months after the beginning of treatment, presented higher CXCL10, CCL22/Macrophage-Derived Chemokine (MDC) and soluble Tie2 plasma levels before initiation of treatment than patients without DCB (Fig. 3A). Flow cytometry analyses on fresh whole blood highlighted that CD4^+^ PD1^+^ OX40^+^ and CD4^+^ α4β7^+^ among total CD4^+^ T cells were more present in patients with DCB than patients without DCB (Fig. 3B). IHC analyses on pre-treatment tumor biopsies highlighted that patient who developed DCB tended to have higher immune infiltration characterized by higher percentage of PDL1^+^ tumor cells, and higher densities of CD3^+^ T cells, FOXP3^+^ cells and DCLAMP^+^ dendritic cells than patients without DCB after treatment (Fig. 3C).

### Specific angiogenic and immune changes occurring during nintedanib lead-in monotherapy could favor primary resistance to immune checkpoint blockade efficiency

During the first week of the clinical trial, patients received nintedanib monotherapy. The comparison of soluble factors in plasma and phenotype of fresh blood circulating lymphocytes between day - 7 and C1D1 allowed the identification of changes associated with DCB to treatment which could be induced by the lead-in nintedanib monotherapy.

**Fig. 4:**
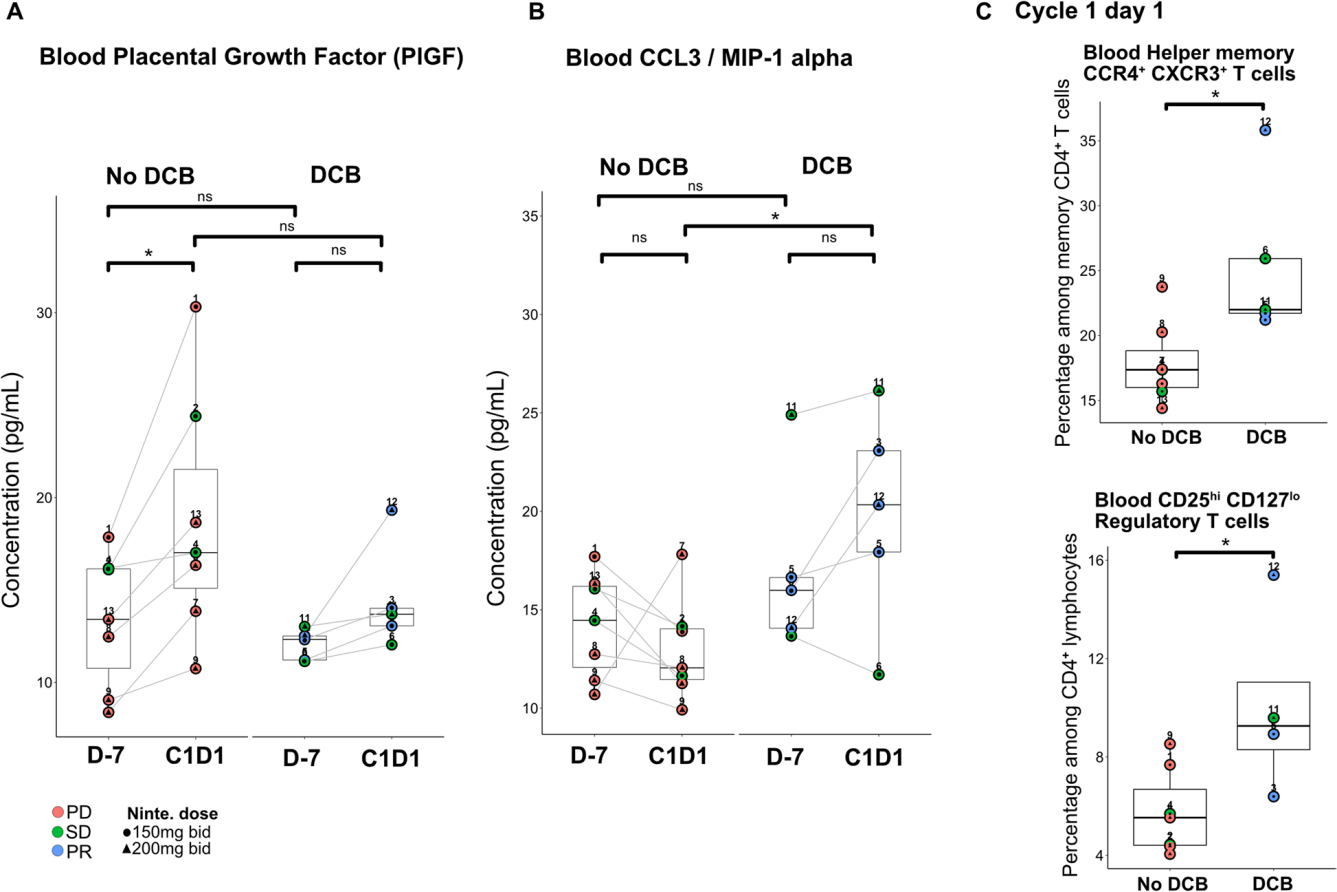
Increased of circulating soluble PlGF, CCL3 and increase in specific T cells subsets during lead-in nintedanib monotherapy associated to clinical outcomes. **A** Plasma rate of Placental Growth Factor (PlGF) increased in patients without DCB between D-7 (13.4 pg/mL [range: 8.4–17.9]) and C1D1 (18.8 pg/mL [range: 10.8–30.3]) (Paired Wilcoxon signed rank test; *p* = 0.015). **B** Plasma rate of CCL3 were higher in patients with DCB (19.8 pg/mL [range: 11.7–26.1]) than without DCB (12.9 pg/mL [range: 9.9–17.8]) after 7 days of nintedanib monotherapy (non-parametric Wilcoxon rank sum test; *p* = 0.03). **C** Percentage of blood CCR4^+^ CXCR3^+^ T cells among effector memory CD4+ T cells and CD25^high^ CD127^low^ Tregs among total CD4+ T lymphocytes were higher at C1D1 in patients with DCB (25.3% [range: 21.2–35.8] and 10% [range: 6.4–15.4], respectively) than those without DCB (17.9% [range: 14.4–23.8] and 5.8% [range: 4.1–8.5], respectively) (non-parametric Wilcoxon rank sum test; *p* = 0.017 and *p* = 0.024, respectively). *Abbreviations: PD = Progressive disease; SD = Stable disease; PR = Partial response; Ninte. = nintedanib; DCB = Durable clinical benefit; D-7 = day − 7; C1D1 = Cycle 1 day 1; MIP = Macrophage Inflammatory Protein*

Placental Growth Factor (PlGF) and VEGF-D plasma levels increased significantly between day − 7 and C1D1 only in patients without DCB (Fig. 4A; Supplementary Fig. 1), although other soluble angiogenic factors levels remained stable. In parallel, plasma levels of CCL3 / MIP-1α (Macrophage Inflammatory Protein 1-Alpha), another ligand for CCR4 with CCL22/MDC, tended to increase in patients with DCB and was significantly higher before pembrolizumab infusion in patients with DCB compared to patients without DCB (Fig. 4B). Also, patients who benefited from treatment had a higher percentage of circulating conventional CCR4^+^ CXCR3^+^ helper memory T cells and Tregs than patients without DCB (Fig. 4C).

### Early inflammatory changes occurring after immune checkpoint blockade were associated with resistance to treatment and progressive disease

**Fig. 5:**
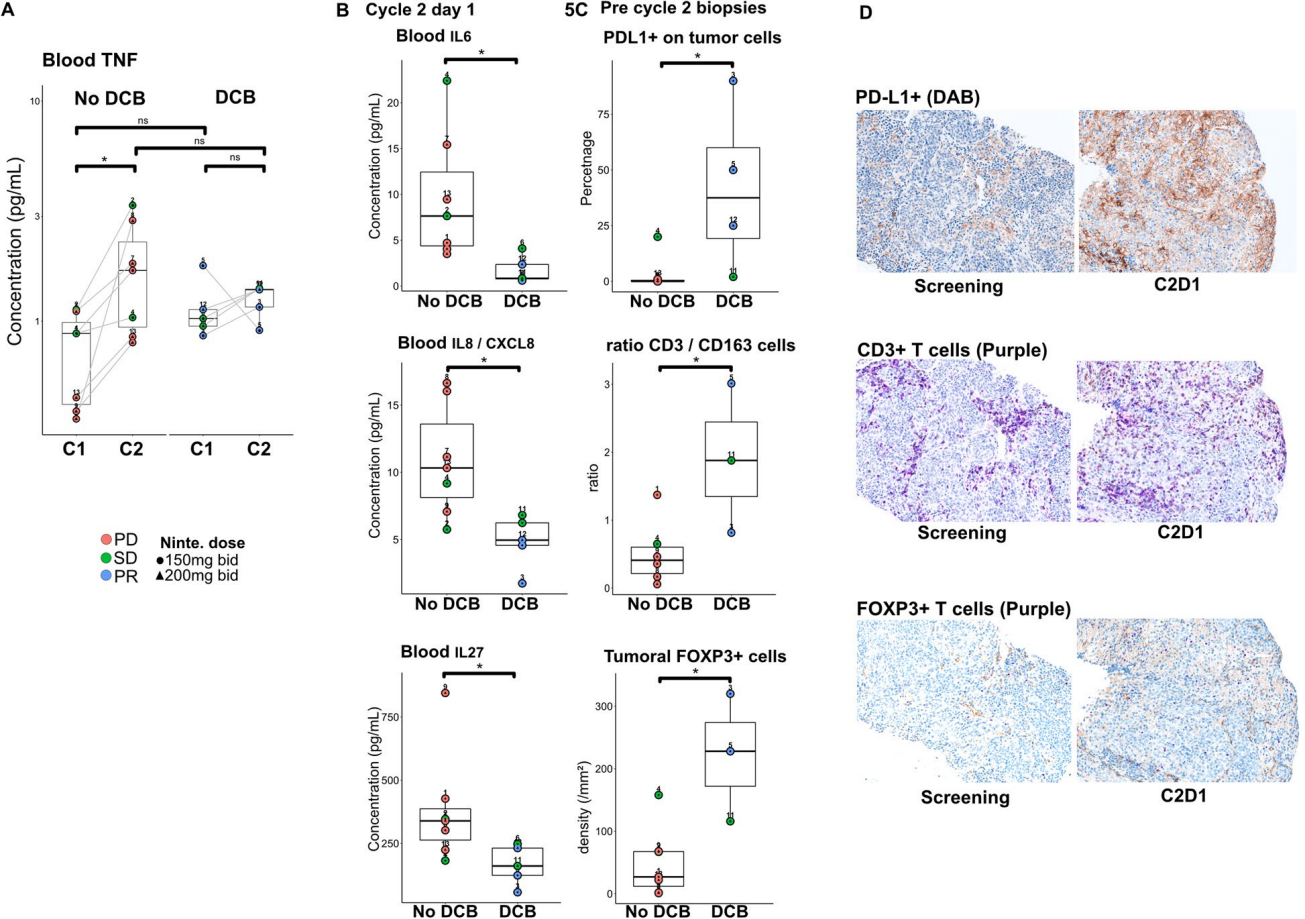
After pembrolizumab infusion, in association with nintedanib treatment, circulating and tumor microenvironment changes were associated with distinct clinical outcomes. **A** Plasma levels of soluble TNF increased in patients without DCB between C1D1 (0.7 pg/mL [range: 0.4–1.1]) and C2D1 (1.8 pg/mL [range: 0.8–3.4]) (Paired Wilcoxon signed rank test; *p* = 0.015) instead of in patients with DCB. **B** Plasma rate of IL6, IL8 and IL27 were significantly higher at C2D1 in patients without DCB (9.6 pg/mL [range: 3.5–22.4], 10.8 pg/mL [range: 5.8–16.7] and 380.9 pg/mL [range: 181.8–845.2], respectively) than those with DCB (1.7 pg/mL [range: 0.6–4.14], 4.9 pg/mL [range: 1.7–6.8] and 163.9 pg/mL [range: 56.6–247.4], respectively) (non-parametric Wilcoxon rank sum test; *p* = 0.01, *p* = 0.01 and *p* = 0.03, respectively). **C** Percentage of PDL1+ tumor cells, ratio of CD3+ per CD163+ cells and FOXP3+ cells density in biopsies of patients with DCB (41.8% [range: 2–90], 1.9 [range: 0.8–3] and 221/mm^2^ [range: 116–320], respectively) was higher than patients without DCB (3% [range: 0–20], 0.5 [range: 0.06–1.4] and 49/mm^2^ [range: 1–158], respectively) at C2D1 (non-parametric Wilcoxon rank sum test; *p* = 0.009, *p* = 0.047 and *p* = 0.03, respectively). **D** Illustration of increased expression of PDL1 at tumor cell’s surface, CD3+ and FOXP3+ T cells infiltration in tumor microenvironment observed with IHC analysis (patient #3). *Abbreviations: PD = Progressive disease; SD = Stable disease; PR = Partial response; Ninte. = nintedanib; DCB = Durable clinical benefit; C1 = Cycle 1; TNF = Tumor Necrosis Factor*

Early inflammatory changes were observed in plasma of patients without DCB as opposed to patients with DCB. Before the second pembrolizumab infusion, patients without DCB presented an increasing plasmatic rate of TNF, compared to paired pre-pembrolizumab plasma (Fig. 5A), and also for CCL3, CCL4, IL-18, IL-10, IL-22 and VEGF-D (Supplementary Fig. 2). Patients without DCB also had higher plasmatic rate of inflammatory cytokines such as IL-6, IL-27 and CXCL8 than patients with DCB at C2D1 (Fig. 5B). IHC performed on tumor biopsies done before C2D1, showed that percentages of PDL1^+^ tumor cells, ratio of CD3^+^ on CD163^+^ cells and density of FOXP3^+^ cells were significantly higher in patients with DCB than without DCB (Fig. 5C). Tumor densities of CD68^+^ and CD163^+^ cells were not different in C2D1 biopsies (Supplementary Fig. 3); neither those of DC-LAMP^+^, IDO1^+^, ICAM1^+^ or CD31^+^ cells (data not shown).

## Discussion

This phase 1b dose escalation cohort showed that the toxicity of the nintedanib and pembrolizumab combination was manageable and consistent with the safety profile of each drug and did not seem to generate higher toxicity than the cumulative impact of each compound. The RP2D for the combination was defined as nintedanib 150 mg bid and flat dose of pembrolizumab 200 mg IV and is currently being investigated in multiple expansion cohorts. The main toxicity of this combination therapy was liver enzymes increase, which is consistent with other combination of immune checkpoint inhibitors and antiangiogenic TKIs. The incidence of drug-related grade 3 liver enzymes elevation was 33% for alanine aminotransferase and 25% for aspartate aminotransferase in our study. Other combinations of anti PD-1 monoclonal antibody and antiangiogenic TKI reported variable liver toxicity: nivolumab/sunitinib, nivolumab/pazopanib, pembrolizumab/pazopanib and pembrolizumab/axitinib reported 18, 20%, 60–70 and 8% of grade 3–4 elevated alanine aminotransferase, respectively [17, 25, 26]. In the dose escalation trial of nintedanib monotherapy, the DLTs were grade 3 or 4 liver enzyme elevations (2–5%), all reversible upon drug interruption [27]. The adverse events reported in this study seem to be mostly related to nintedanib but programmed death 1 blockade could possibly enhance the on-target off-tumor side effects of the TKIs. The observed levels of immune related adverse events such as colitis (8%), nephritis (8%) and thyroiditis (25%) were in line with the expected irAE associated with pembrolizumab monotherapy. Thromboses were observed in two patients: one with superior mesenteric artery thrombus and both with thrombus on catheter in superior vena cava. These adverse events could be related to the combination of antiangiogenic TKI with immune checkpoint blockade and not to nintedanib alone. These two patients with thromboses obtained a durable clinical benefit (SD and PR, respectively during at least 6 months). Overall, the antitumor activity of the combination tested was promising with an ORR of 25% in this all-comer cohort and will be confirmed in the expansion cohorts of the study.

Baseline ancillary analyses highlighted those patients who developed DCB to this anti-angiogenic / ICB combination presented with both preexisting immune and angiogenic features. Higher expression of CXCL10 and immune infiltrate in biopsies were previously associated with ICB monotherapy efficiency in different tumor types [28, 29]. It was interesting to observe that soluble Tie2 was higher in plasma of patients with DCB. Tie2 is the receptor of angiopoietin-2, expressed at surface of endothelial cells but also circulating monocytes [30]. Studies highlighted that Tie2 production and membrane shedding are increased by hypoxia and induced by VEGF [31]. Here, Tie2 plasmatic rate could be a witness of hypoxia occurring in the tumor of patients who benefited from the treatment.

After seven days of nintedanib monotherapy, quick changes were observed in patients who benefited from the treatment. Although those could be spontaneous changes favored by different tumor changes, their rapid evolution suggest that they could be induced by nintedanib. Particularly, the increase of plasmatic CCL3/MIP-1α in patients with DCB could be a consequence of tumor hypoxia on chemokine production [32]. At baseline, a higher Tie2 plasmatic rate, along with higher proportions of circulating Tregs, and conventional helper memory T cells which co-expressed CCR4 and CXCR3 (receptors of CCL22/MDC, CCL3/MIP-1α and CXCL10 respectively) were also predictive of DCB. Blood Tregs constitutively express CCR4 on their surface [33]. CCL22 production by tumor cells was identified as a mechanism of immune escape in breast cancer, through CCR4^+^ Treg recruitment in the tumor microenvironment (TME), induced by IFNγ exposure [34, 35]. These observations led to the development of CCR4 antagonists as antitumoral immunotherapy [36, 37]. Our results suggest that Treg recruitment in the TME was associated with a higher infiltration of immune cells, particularly DC-LAMP^+^ dendritic cells and T cells, associated with a pre-existing anti-tumoral immune response, sensitive to immune checkpoint blockade.

However, patients without DCB developed others significant changes after one week of nintedanib monotherapy. They seemed to present an increasing plasma rate of PlGF as of patients with DCB. This soluble angiogenic factor was recently implicated in interactions between angiogenesis, Th17 polarization of CD4^+^ T cells and autoimmunity [38]. This observation should be associated with changes induced further by anti PD-1 blockade. Patients with tumor progression and resistance to treatment presented then with an increasing plasma rate of IL17A and TNF after the first infusion of pembrolizumab, in comparison to patients with DCB. Patients without DCB had also a higher plasma rate of IL-6, IL-8/CXCL8 and IL-27 at C2D1 than patients who had benefited from treatment. Increased rates of both IL-6 and IL-8/CXCL8 have been described to be associated with resistance to anti-PD1 blockade monotherapy [39, 40]. Of note, the baseline plasma rates of these cytokines were not significantly different between patients with or without DCB outcome.

Interestingly, VEGF-D levels were significantly increasing in patients with No DCB upon nintedanib monotherapy (between D-7 and D1; Supplementary Fig. 1) and upon addition of pembrolizumab (between C1D1 and C2D1; Supplementary Fig. 2). Patients with DCB did not show a specific change in VEGF-D levels upon treatment. This observation raises the question of potential differential sensitivity to angiogenic inhibitors between patients presenting DCB and No DCB via either variations in target receptor expressions or reactional feedback loops.

## Conclusion

Nintedanib 150 mg bid is the RP2D for combination with pembrolizumab and is being currently investigated in multiple expansion cohorts. Early immune parameters in the tumor and in the blood were associated with the efficacy of the nintedanib + pembrolizumab combination therapy. Our biological findings at baseline and during the early phases of treatment, associated significantly with the clinical benefit to treatment or to primary tumor progression, suggesting that patients could be selected on their tumor biology rather than their cancer histology in order to benefit from such anti-angiogenic and immune targeted therapies.

## Supplementary Information

**Additional file 1: Supplementary Fig. 1.** Evolution of plasma soluble angiogenic factors during lead-in nintedanib monotherapy. Tests were paired Wilcoxon signed rank test (paired samples) (representation of *p*-value: ns > 0.05, * ≤ 0.05). Abbreviations: Ninte. = nintedanib; PD = Progressive disease; SD = Stable disease; PR = Partial response; DCB = Durable clinical benefit; D-7 = day − 7; C1D1 = Cycle 1 day 1.

**Additional file 2: Supplementary Fig. 2.** Evolution of plasma soluble cytokines between after the first pembrolizumab infusion, between cycle 1 and cycle 2. Tests were paired Wilcoxon signed rank test (paired samples) (representation of p-value: ns > 0.05, * ≤ 0.05). Abbreviations: PD = Progressive disease; SD = Stable disease; PR = Partial response; Ninte. = nintedanib; DCB = Durable clinical benefit; D-7 = day − 7; C1D1 = Cycle 1 day 1; MIP = Macrophage Inflammatory Protein; MCP1 = Monocyte Chemoattractant protein; MDC = Macrophage Derived Chemokine.

**Additional file 3: Supplementary Fig. 3.** Illustration of multiplex chromogenic staining dedicated to myeloid cells. **A** Representative image displays a 500umx669um image after multispectral imaging and spectral unmixing (merged image). **B** All markers. **C** CD68 (pseudocoloured white). **D** DAPI nuclear marker (pseudocoloured blue). **E** CD163 (pseudocoloured green). **F** DC-LAMP (pseudocoloured magenta). **G** IDO1 (pseudocoloured yellow). **H** CD163 measurement was undertaken using Inform v2.2 software segmentation by pixel-based threshold. **I** CD163^+^ density in biopsies of patients with DCB or without DCB at baseline and C2D1 were not significantly different (Wilcoxon rank-sum test).

## Data Availability

The datasets used and/or analyzed during the current study are available from the corresponding author on reasonable request.

## Abbreviations

ANSM: Agence Nationale de Sécurité du Médicament
DCB: Durable clinical benefit
DL1: Dose level 1
DLT: Dose-limiting toxicity
ECOG: Eastern Cooperative Oncology Group
FGF: Fibroblast growth factor
HES: Hematoxylin and Eosin Staining
HCC: Hepatocellular carcinoma
IHC: Immuno-Histo-Chemistry
irRECIST: Immune-related RECIST
MDC: Macrophage-Derived Chemokine
MIP-1α: Macrophage Inflammatory Protein 1-Alpha
MTD: Maximal tolerated dose
NCI: National Cancer Institute
NSCLC: Non-small cell lung cancer
PD: Progressive disease
PDGF: Platelet-derived growth factor
PD-1: Programmed Death-1
PlGF: Placental Growth Factor
Po: Per os
PR: Partial response
RCC: Renal cell carcinoma
RECIST: Response Evaluation Criteria in Solid Tumors
RP2D: Recommended Phase 2 Dose
SD: Stable disease
UCNT: Undifferentiated carcinoma of nasopharyngeal type
VEGF: Vascular Endothelial Growth Factor
