# Supplementary figures and images for: Safety, recommended dose, efficacy and immune correlates for nintedanib in combination with pembrolizumab in patients with advanced cancers
Source: J Exp Clin Cancer Res. 2022 Jul 7;41:217. doi: 10.1186/s13046-022-02423-0 (PMC9260998; doi:10.1186/s13046-022-02423-0)

Suppl Fig 1

● PD Ninte. dose  
● SD 150mg bid  
● PR 200mg bid

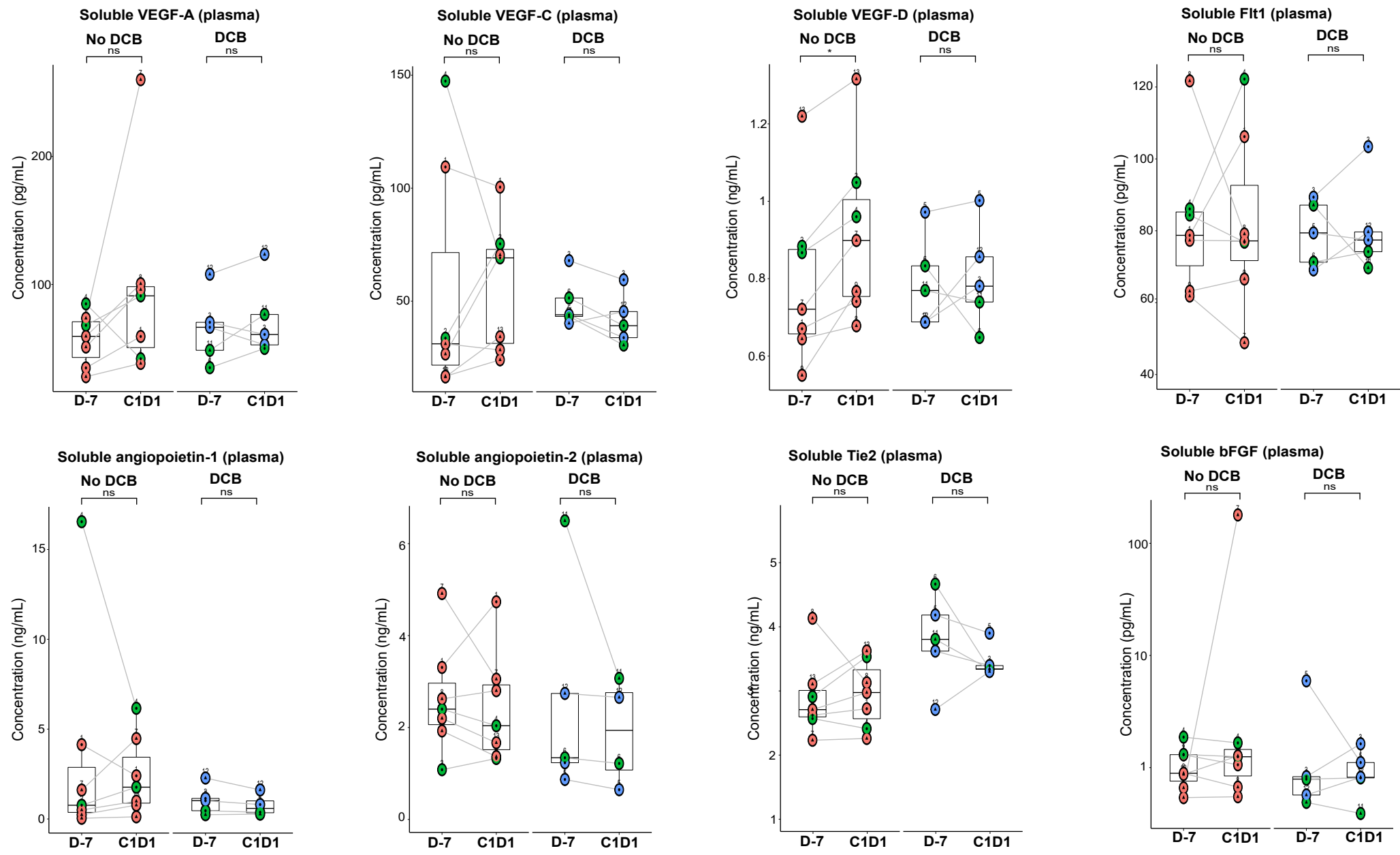

Supplement: Supplementary file 1 — Additional file 1: Supplementary Fig. 1. Evolution of plasma soluble angiogenic factors during lead-in nintedanib monotherapy. Tests were paired Wilcoxon signed rank test (paired samples) (representation of p-value: ns > 0.05, * ≤ 0.05). Abbreviations: Ninte. = nintedanib; PD = Progressive disease; SD = Stable disease; PR = Partial response; DCB = Durable clinical benefit; D-7 = day − 7; C1D1 = Cycle 1 day 1. [file 13046_2022_2423_MOESM1_ESM.pdf]

Suppl Fig 2

● PD Ninte. dose  
● SD ●150mg bid  
● PR ▲200mg bid

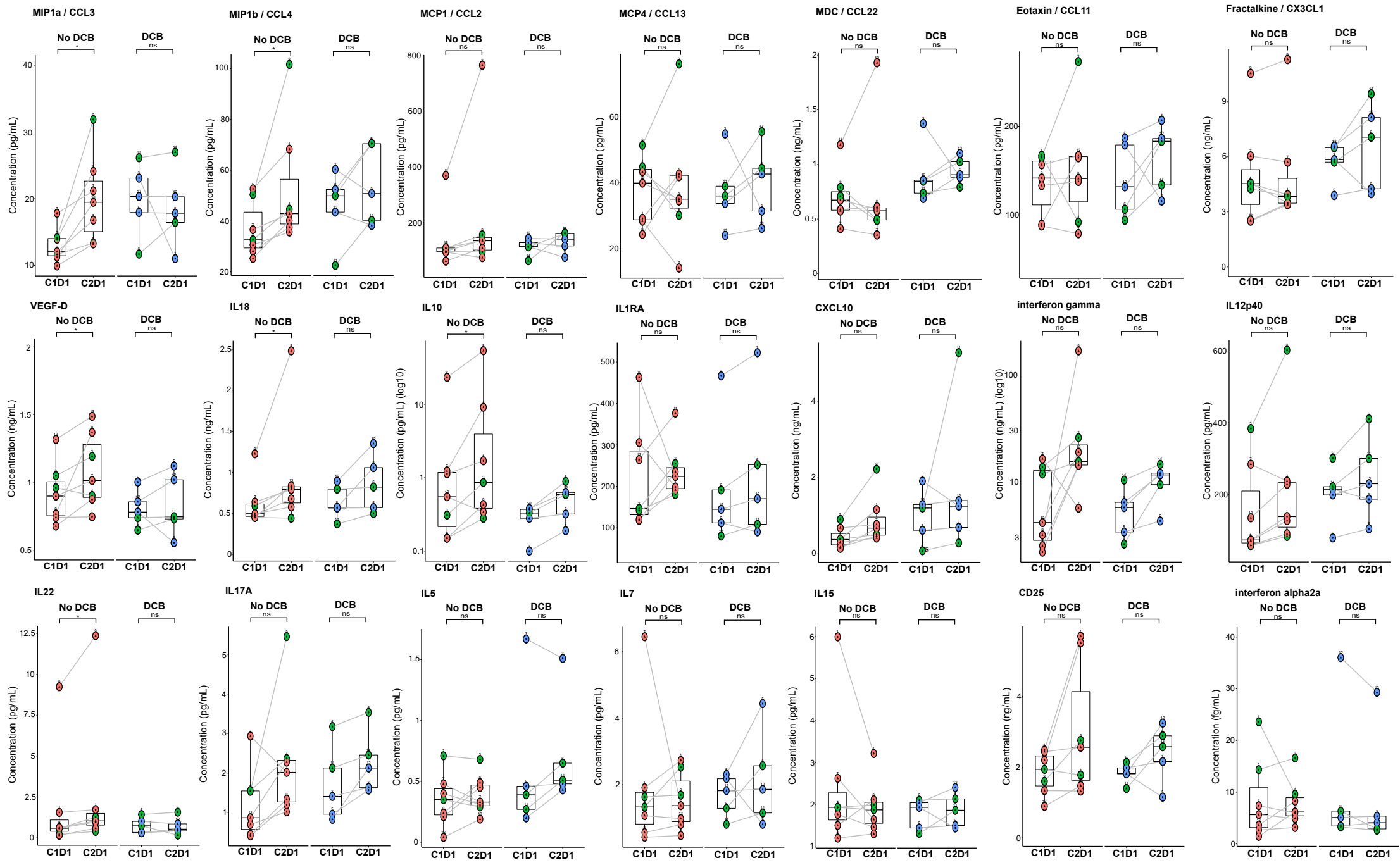

Supplement: Supplementary file 2 — Additional file 2: Supplementary Fig. 2. Evolution of plasma soluble cytokines between after the first pembrolizumab infusion, between cycle 1 and cycle 2. Tests were paired Wilcoxon signed rank test (paired samples) (representation of p-value: ns > 0.05, * ≤ 0.05). Abbreviations: PD = Progressive disease; SD = Stable disease; PR = Partial response; Ninte. = nintedanib; DCB = Durable clinical benefit; D-7 = day − 7; C1D1 = Cycle 1 day 1; MIP = Macrophage Inflammatory Protein; MCP1 = Monocyte Chemoattractant protein; MDC = Macrophage Derived Chemokine. [file 13046_2022_2423_MOESM2_ESM.pdf]

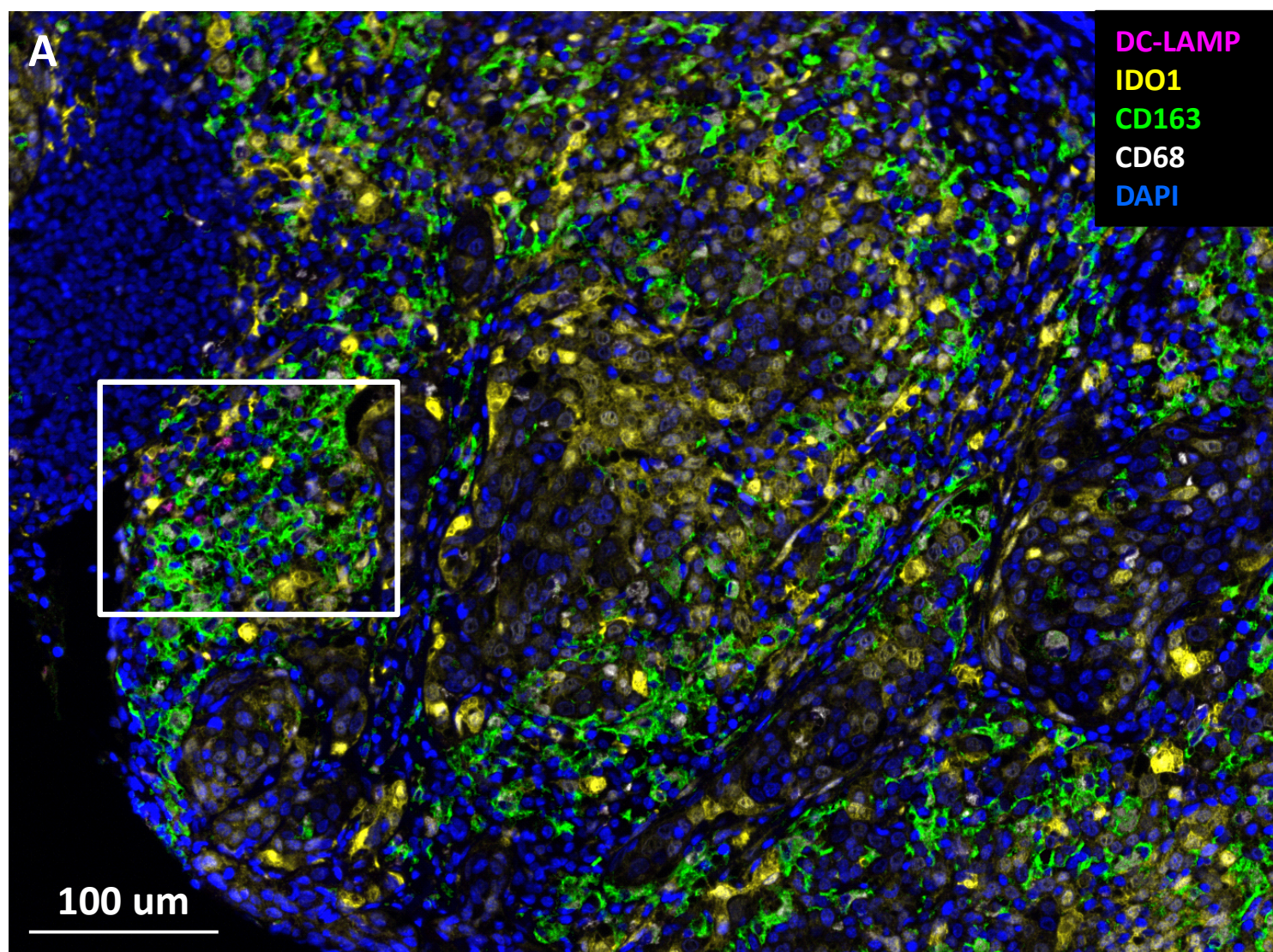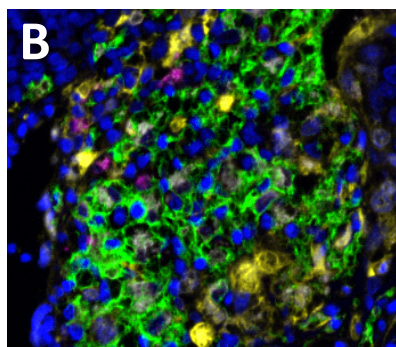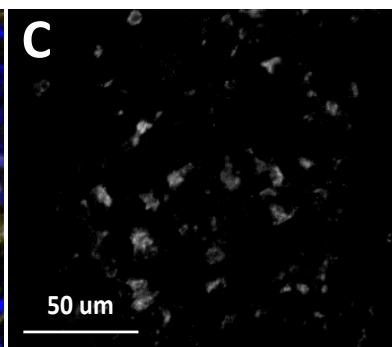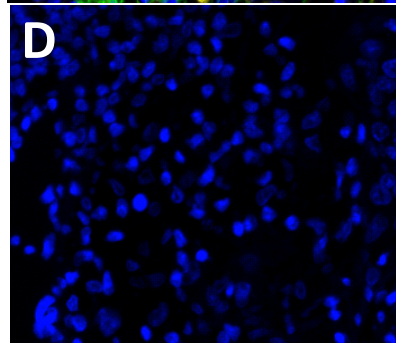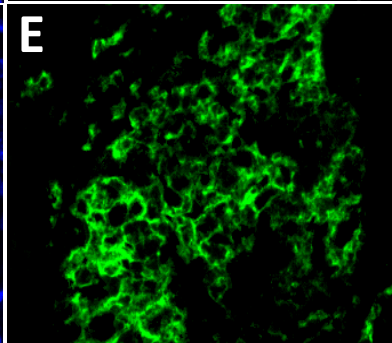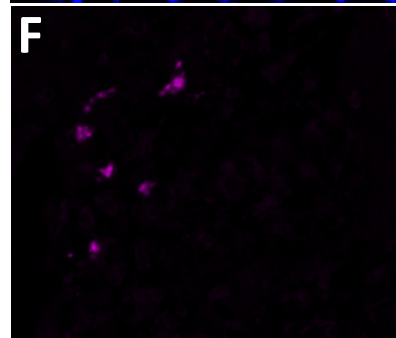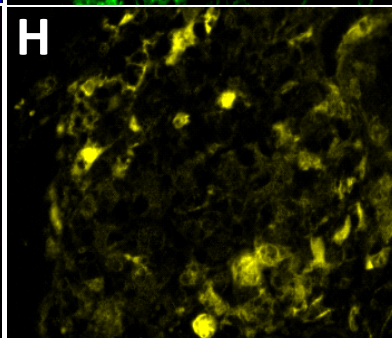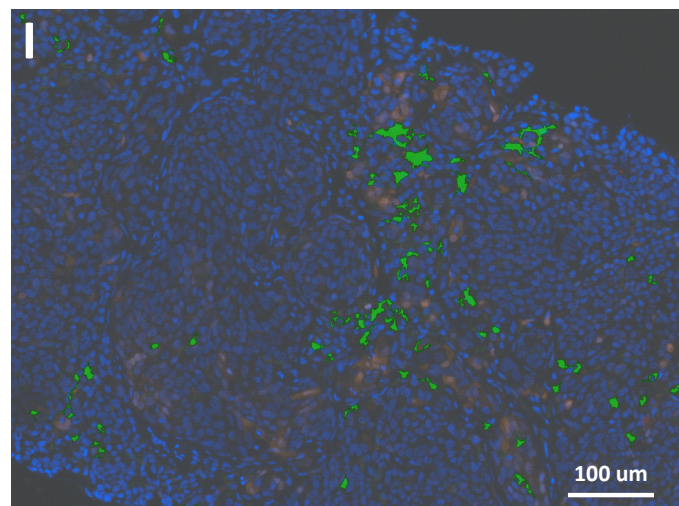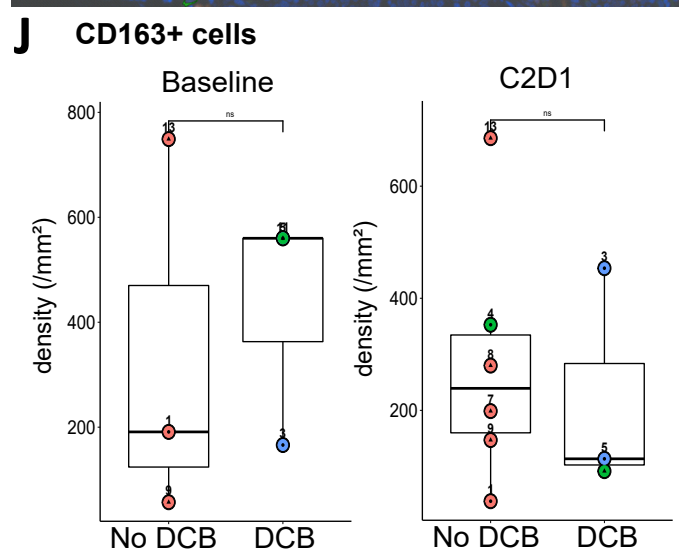

Supplement: Supplementary file 3 — Additional file 3: Supplementary Fig. 3. Illustration of multiplex chromogenic staining dedicated to myeloid cells. A Representative image displays a 500umx669um image after multispectral imaging and spectral unmixing (merged image). B All markers. C CD68 (pseudocoloured white). D DAPI nuclear marker (pseudocoloured blue). E CD163 (pseudocoloured green). F DC-LAMP (pseudocoloured magenta). G IDO1 (pseudocoloured yellow). H CD163 measurement was undertaken using Inform v2.2 software segmentation by pixel-based threshold. I CD163+ density in biopsies of patients with DCB or without DCB at baseline and C2D1 were not significantly different (Wilcoxon rank-sum test). [file 13046_2022_2423_MOESM3_ESM.pdf]
